# Withaferin A—A Promising Phytochemical Compound with Multiple Results in Dermatological Diseases

**DOI:** 10.3390/molecules26092407

**Published:** 2021-04-21

**Authors:** Simona Bungau, Cosmin Mihai Vesa, Areha Abid, Tapan Behl, Delia Mirela Tit, Anamaria Lavinia Purza, Bianca Pasca, Laura Maghiar Todan, Laura Endres

**Affiliations:** 1 Department of Pharmacy, Faculty of Medicine and Pharmacy, University of Oradea, 410028 Oradea, Romania; dtit@uoradea.ro (D.M.T.); apurza@uoradea.ro (A.L.P.); biancapasca28@yahoo.com (B.P.); 2 Doctoral School of Biomedical Sciences, University of Oradea, 410073 Oradea, Romania; lauratodan@yahoo.ro; 3 Department of Preclinical Disciplines, Faculty of Medicine and Pharmacy, University of Oradea, 410073 Oradea, Romania; v_cosmin_15@yahoo.com; 4 Department of Food Science, Faculty of Agricultural and Food Sciences, University of Debrecen, 4032 Debrecen, Hungary; areha.abid786@gmail.com; 5 Department of Pharmacology, Chitkara College of Pharmacy, Chitkara University, Punjab 140401, India; tapanbehl31@gmail.com; 6 Department of Psycho-Neuroscience and Recovery, Faculty of Medicine and Pharmacy, University of Oradea, 410073 Oradea, Romania

**Keywords:** *Withania somnifera*, withaferin A, dermatology, dermatological disorders, melanoma, Ayurvedic medicine

## Abstract

Withaferin A (WFA) was identified as the most active phytocompound of the plant *Withania somnifera* (*WS*) and as having multiple therapeutic/ameliorating properties (anticancer, antiangiogenic, anti-invasive, anti-inflammatory, proapoptotic, etc.) in case of various diseases. In drug chemistry, WFA in silico approaches have identified favorite biological targets, stimulating and accelerating research to evaluate its pharmacological activity—numerous anticancer effects manifested in various organs (breast, pancreas, skin, colon, etc.), antivirals, anti-infective, etc., which are not yet sufficiently explored. This paper is a synthesis of the most relevant specialized papers in the field that are focused on the use of WFA in dermatological diseases, describing its mechanism of action while providing, at the same time, details about the results of its testing in in vitro/in vivo studies.

## 1. Introduction

Dermatological diseases are a wide and diverse class of diseases that range from simple rashes to severe skin infections, which may have various causes as follows: allergens, heat, disorders of some systems, drug therapies, etc. They affect about 30% of the population, with the most serious consequences on the quality of life or could even lead to death, causing physical discomfort, embarrassment, the patient sometimes suffering socio-professional restrictions. Moreover, chronic conditions lead to the need for repeated sick leave, with financial repercussions on the patients [1].

*Withania somnifera* (L.) Dunal (*WS*) is a largely used medicinal plant (known also under the name Ayurvedic Ashwagandha) with easily recognizable anticarcinogenic importance and role, leaves of the shrub being used as raw material for the extraction of withanolide steroid compounds [2,3]. This Ayurvedic herb, known across the world for its many beneficial health care practices and roles since ancient times [4], is a member of the *Solanaceae* family. It offers therapeutical effects for many human diseases, including arthritis, epilepsy, depression, diabetes, and has palliative effects (such as analgesics, growth promoting, regenerating, rejuvenating, etc.). In patients suffering from the above-mentioned disorders, some clinical studies focused on the various sections/parts of the herb that have shown protection and have proved to be safe [5].

**Figure 1:**
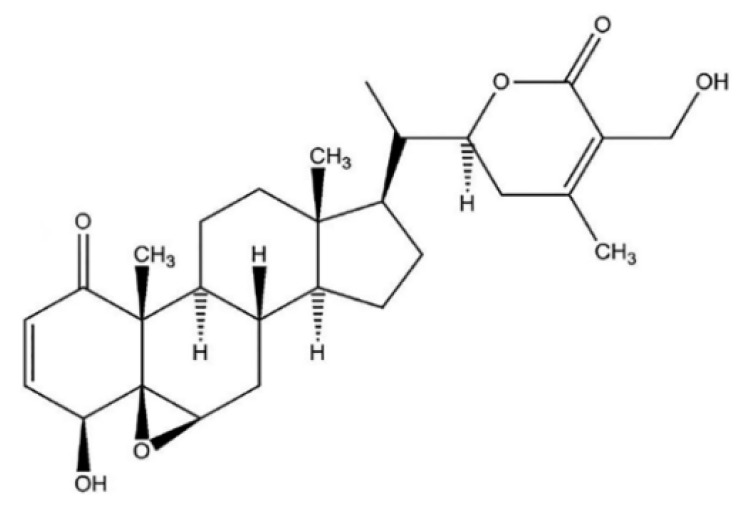
WFA chemical structure.

Withaferin A (WFA) was the first isolated, most common, and frequently identified withanolide in *WS* [6], with a detailed chemical structure depicted in Figure 1.

Worldwide, several studies were conducted on the WFA properties and on its beneficial action in anticancerous therapy. Nowadays, there are numerous published data on the antitumoral action of WFA, performed by making use of different techniques (xenografts, orthotopic tumor models, and cell cultures of multiple human cancers) [3,7]. Many of these studies revealed the cytotoxic, antimigratory, proapoptotic, and anti-invasive actions of WFA, in the case of different types of cancers. Various molecular targets in cancerous cells are modulated by WFA, especially signaling molecules, enzymes, and various proteins that are essential for the survival of tumor cells [8,9]. WFA may also operate through processes such as triggering proteinase-activated receptor 4 (PAR-4) and suppressing heat shock protein 90 (HSP90) in prostate cancer cells [10,11], obstructing nuclear factor kappa-light-chain enhancer of activated B cells (NF-κB) [12], triggering deoxyribonucleic (DNA) acid damage checkpoint (G2-M) inhibition; forkhead box O3 (FoxO3a) and apoptotic protein Bim adjustment in breast cancer [13].

Through the intrinsic or extrinsic routs, WFA was indicated to trigger apoptosis in the human breast, prostate, neck and head, leukemia, and melanoma tumor cells by stimulating several proteases and caspases and decreasing mitochondrial membrane voltage, which induces damage of several substrates such as poly adenosine diphosphate (ADP)-ribose) polymerase cleavage and cytoskeletal proteins [14,15,16].

Moreover, WFA targets some proteins resulted from the anti-stress pathway, implying the improvement and enhancement in reactive oxygen species (ROS) expression. The increased ROS amount also enables antioxidant pathways and causes disequilibrium in ROS/cytoprotection action, determining the cancer cells stage. WFA therapy activates four stress response proteins for oxidative damage reduction and restores homeostasis during and after the treatment. The WFA therapy upregulates proteins such as aldose reductase, heme oxygenase, iron–sulfur, and sepiapterin reductase as a response to oxidative stress, while glutathione peroxidase 1, hydroperoxide, and phospholipids are downregulated [17,18].

Two studies demonstrated that the nuclear factor erythroid 2–related factor 2 (Nrf2) is suitable for stripping oxidized protein and maintaining homeostasis following oxidative stress and ubiquitin–proteasome (UPS) activations. Targeted UPS causes more proteotoxic stress in the cancer-type cells. UPS-related WFA therapy upregulates five proteins as follows: beta-1 (human PSB1), alpha-2 subunit proteasome (human PSA2), 10B subunit (regulatory) of 26S proteasome, UBP24 carboxy-terminal hydrolase of ubiquitin (human), and subunit-4 complex activator of the proteasome (PSME4 human). Among the WFA-target proteins, there are considered degraded proteins, adenylpyrophosphatase associated with various activities (AAA+) chaperone p97, proteasome beta-types 10 and 5, and human isozyme L5 (USP25) [19,20].

This paper is a synthesis of the most relevant specialized published data in the field focused on the use of WFA in some of the most frequent and known dermatological diseases (skin cancers, pigmentation disorders, scleroderma, herpes simplex, etc.). Moreover, its novelty character consists precisely in this focus of the topic on the use of WFA in skin disorders since there is no study of this type so far, following all the investigations we have performed. The in-depth research presents, in a systematized way, all the general and special aspects on the topic of WFA actions and their role in dermatology. Thus, our review results in an extremely solid basis for further studies in the research area addressed below, suggesting new ideas of study related to the use of this phytochemical compound or the possibility of its testing in clinical trials.

## 2. Methodology

**Figure 2:**
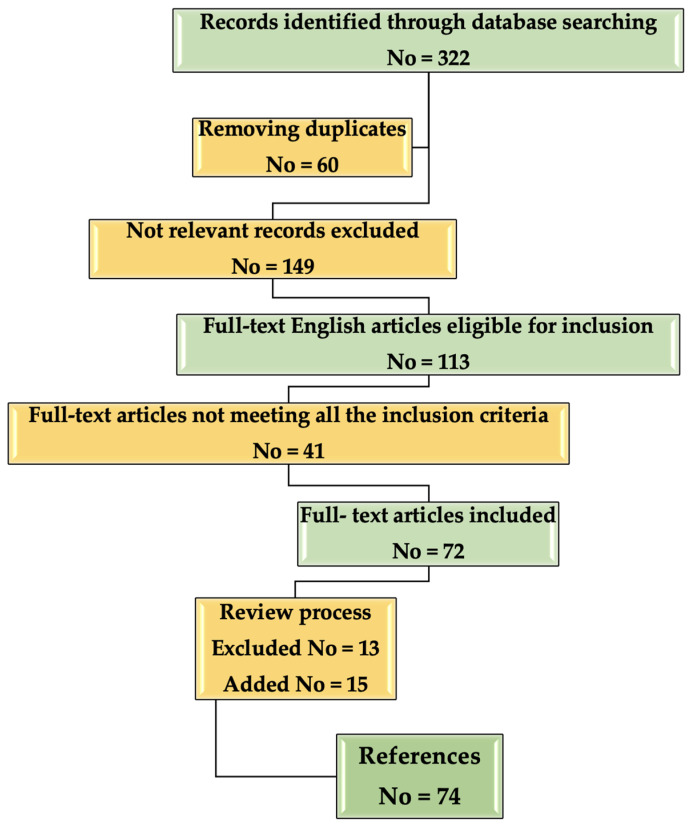
Flow chart describing the selection process of the References included in the study.

A flow chart about the inclusion/selection criteria of the papers that were considered for this research is presented in Figure 2.

## 3. Withaferin in Skin Cancers

Skin cancer is known to be caused by uncontrolled growth of abnormal epidermal cells (epidermis is the outer layer of the skin), as a result of irreparable DNA damage, a fact that inevitably leads to mutations. Skin cancers are classified into the following main types—basal cell carcinoma (BCC), squamous cell carcinoma (SCC), melanoma, and Merkel cell carcinoma, all of them among the most common cancers worldwide [21,22].

The most lethal cutaneous neoplasm is considered to be malignant melanoma. Achieved drug resistance commonly appears sometime after concrete tumor response, explaining the necessity for new remedies [23,24].

WFA was found to inhibit in vivo mouse melanoma (B16F1) tumor development [25]. For a different heterograft skin cancer type operating with 92.1 uveal melanoma cells, following the treatment with WFA, approximately 29% of mice presented total clinical reaction, whereas 43% of the subjects indicated cancer evolution when the treatment was interrupted [22]. As other recent published data revealed, WFA decreased the tumor abundance; however, it did not reduce the prevalence of 12-*O*-tetradecanoylphorbol-13-acetate (TPA) favored and dimethyl-benzanthracene DMBA-induced mice skin cancer development [26], in part by triggering activator protein-1 (AP-1) and inhibiting the expression of acetyl coenzyme A carboxylase-1 (ACC1) [27,28]. Chemopreventive and antitumor effects of WFA, as well as the mechanisms involved and its use as supportive therapy in skin cancers, are analyzed in the following subsections.

### 3.1. Chemopreventive Effects

Some studies indicated the chemopreventive capacity of *WS* that can be useful against skin carcinogenesis [29,30].

WFA has a thoroughly analyzed antitumoral function in experimental animal models of human cancer; however, it has uncertain chemopreventive capacity. Published data have shown that in the well-established tumor promotion model for JB6 P+ skin epidermal cells WFA suppressed the cell transformation and proliferation induced by the tumor promoter TPA [27]. It is interesting that TPA deactivated isocitrate dehydrogenase 1 (IDH1), reversed by WFA. In mouse skin tissue, similar findings were presented. Thus, metabolism was considered as the possible mechanism of action. It was found that mitochondrial activities such as membrane voltage, complex I action, and mitochondrial breathing have been decreased by TPA therapy. Nevertheless, WFA restrained all these downregulations. Moreover, α-ketoglutarate levels, an IDH1 product, were investigated and WFA inhibited its decrease in TPA therapy. Furthermore, the lactate level was observed as an indicator for glycolysis, WFA inhibiting its increase due to treatment with tumor promoters [26].

In 2019, Xu et al. [31] studied the way WFA inhibited the IDH1 favored skin cancer. Data obtained revealed the WFA ability to stabilize IDH1 by suppressing the ubiquitin-proteasome pathway (UPP). As a result of clarifying the process of IDH1 suppressing carcinogenesis, the outcomes reflected that the activity on LDH was inhibited by the increase of IDH1, while the action of mitochondrial complex I was enhanced. Moreover, increased IDH1 determined prolyl hydroxylase (PHD) activation through its product α-ketoglutarate (KG) and suppressed HIF-1α downward signaling route. Findings reveal that WFA partly suppresses cancer evolution through stabilizing IDH1, conducting to the deactivation of HIF-1α signaling [31].

Li et al. [32] indicated that the decrease in tumor multiplicity of DMBA-initiated and TPA favored the development of mouse skin tumors, partially by inhibiting ACC1 expression and triggering the activator protein-1 (AP-1). The chemopreventive ability of WFA was examined in a skin carcinogenesis mouse model, chemically triggered. Pathological studies found that the development of skin tumors was suppressed substantially by WFA. Morphological studies on skin tissue indicate that during skin cancer, WFA inhibited cell growth instead of causing apoptosis [31]. Microarray examination of the antibody showed that WFA suppressed the ACC1 upregulation caused by a carcinogenic agent, which was further verified in a skin cell transformation model. The knockdown of ACC1 inhibited unbundling-independent development and the activation of oncogenic transformation of skin cells, supported by ACC1 overexpression. Further experiments have shown that WFA blocked ACC1 gene transcription caused by tumor promoters, stopping activator protein 1 activation. The expression levels of ACC1 in melanoma cells were also inhibited by WFA. Eventually, research on human skin cancer tissue showed that the ACC1 in tumors was upregulated, compared with neighboring healthy tissues. Results indicate also that WFA may have a chemoprevention capacity, and that ACC1 may represent a key objective for WFA [26].

Summarizing, it can be concluded that chemical-induced skin cancer is inhibited by WFA, and ACC1 can be an effective target for WFA. These data can be extended to melanoma as the expression of ACC1 in melanoma cells is also inhibited by WFA. The study focused on how WFA controls ACC1 and the way ACC1 favors cancer evolution [32].

### 3.2. Antitumor Effects

**Figure 3:**
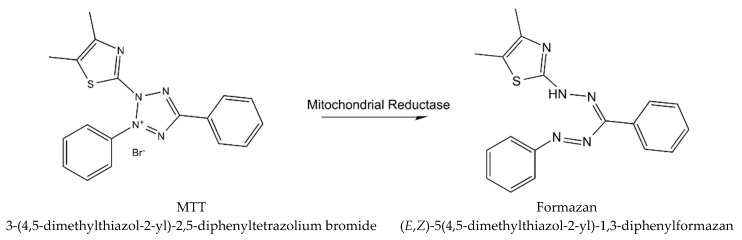
The reaction describing MTT procedure (metabolism of MTT to a formazan salt).

Cytotoxicity of *WS* root mineral extract on A375 (ATCC) human malignant melanoma cell line was tested for the first time in the study of Halder et al. [26]. The extract obtained from blunt *WS* was tested for A375 cell cytotoxicity with the MTT procedure (using yellow tetrazolium MTT reagent, known also as 3-(4,5-dimethylthiazolyl-2)-2, 5-diphenyltetrazolium bromide) [33], which is based on the reaction presented in Figure 3.

The morphology of the treated ATCC has been visualized by phase-contrast and fluorescence microscopy. The deoxyribonucleic acid (DNA) fragmentation of crude extract cells treated with agarose gel electrophoreses was tested. *WS* root crude extract has the ability to minimize the viable dosage and time-dependent cell count. In contrast to untreated or vehicular regulation, morphological changes in the ATCC were also observed for treated types. Under a fluorescence microscope, apoptotic body and nuclear blebbing were detected in the 4′,6-diamidino-2-phenylindole (DAPI) stain-treated cells, allowing a ladder of sequenced DNA observance in the treated cells. Moreover, the fresh aqueous extract of *WS* has a significant cytotoxic effect related to the human malignant ATCC [34].

WFA has a low antitumor and radio-sensitizing function [35]. Published data seek to evaluate the tumor sensitizing effect of WFA on B16F1 melanoma reaction with or without local hyperthermal fractional and acute radiotherapy. The following two series of parameters (each or in association) were described as being used in the adult C57BL mice, injected intradermally with 5 × 10^5^ tumor cells (B16F1 melanoma) on the dorsal skin, with 100 ± 10 mm^3^ tumors: 1. fractionated radiotherapy (RT) (2 Gy × 5 days a week, 4 weeks); WFA (15 mg/kg, 5 days a week, 3 weeks); and local hyperthermia (HT) (43 °C once a week, for 3 weeks) or 2. short-term high-level RT (40 Gy); WFA (40 mg/kg); and HT (43 °C, 30 min). The treatment reaction was evaluated by testing the regression of the tumor, development delay, and animal longevity. In acute RT + HT, 50% of the response was partial, then increased to 62.5% with the WFA mixture. In fractional systems, the mixture of trimodality led to 100% PR. The growth delay (GD) increased with acute RT + HT and WFA + RT in contrast with RT alone that subsequently grew in trimodality therapy. WFA + RT + HT fragmented produced greater GD and survival rates, in a 3-week treatment, higher than other therapies. Finally, in a fractional regimen, HT is less efficient than WFA, which is a better radiosensitizer and facilitates a reduction in radiation exposure for radioresistant tumors such as melanoma by associating the nontoxic doses of WFA with fractional RT, with and without the addition of HT [36].

The apoptotic mechanism initiated by WFA in the melanoma cell lines included mitochondrial translocation, cytochrome c releasing, transmembrane modifications, and activation of caspases 9 and 3. Early reactive oxygen species (ROS) production may also be needed for WFA cytotoxicity [14]. Skin melanoma, which is malignant, highly resistant, and heterogeneous to conventional cancer chemotherapy, constitutes the most aggressive and deadly form of skin cancer. Against several cancer cells, WFA had antitumor action. WFA was first tested in an association of four different melanoma human cells and cellular dynamics research. With IC_50_ varying from 1.8 to 6.1 μM, WFA causes apoptotic cell death. WFA associated Bcl-2/Bax/Bcl-2/Bim apoptosis with low cell ratios. Both lines include the WFA-led mitochondrial-pathway apoptotic process, which was linked to Bcl 2, BaX mitochondrial translocation, cytochrome c release, transmembrane dissipation potential (mm), caspase 9, and DNA fragmentation. In this way, the process is linked with Blu-2 downregulation. WFA cytotoxicity includes the synthesis and depletions of early ROS and ROS inhibitions, leading to the full abolition of mitochondrial and nuclear events by the antioxidant N-acetylcysteine. These effects contribute to WFA therapeutic effect against human melanoma [14].

Histopathological tests on Wistar rats (adult male weighing 125–150 g) cutaneous sample tissue have found enhanced malignancy, with ultraviolet B (UVB) radiation exposure (wavelength of 294 nm) for 20 days, succeeded by benzoyl peroxide topical treatments that promoted tumors (dose of 20 mg/animal/0.2 mL acetone). For animals pretreated with 1-oxo-5- and 6beta-epoxy-with-2-enolide (obtained from *WS* roots) was avoided the occurrence of skin carcinomas when animals were exposed to UVB radiation or to benzoyl peroxide associated with UVB. Administering 1-oxo-5beta or 6beta-epoxy-with-2-enolide also stops malignancy in the skin tissue, following exposure to UVB radiation and benzoyl peroxide. In rats exposed to UVB radiation, immunohistochemical stains of cutaneous tissues reveal p53 + foci (cells that carry the mutant p53 protein), whereas, in animals pretreated with 1-oxo-5beta/6-beta-epoxy-with-2-enolide, the absence of the p53 + foci has been noted. These findings show that the effects on the skin carcinomas caused by UVB radiation are possible in 1-oxo-5beta/6-beta-epoxy-witha-2-enolide [37].

### 3.3. Support Therapy

While previous research studied the impact of WFA on the viability and proliferation of melanoma cells, a thorough analysis on WFA and its therapeutic and concentration interval in which WFA can be used for avoiding side effects was performed using a large melanoma cell line variety and regular fibroblast [28,38]. The B16F1 melanoma and fibrosarcoma, cultivated in C57BL and in Swiss albino mice were tested for the function of WFA, either alone or in combination with fractionated and acute radiotherapy, and/or hyperthermia. Acute dose gamma radiation of 30 or 50 Gy or five fractions of 10 Gy was applied locally on tumors. WFA was injected intraperitoneally, in a dose of 40 mg/kg, 1 h prior to acute radiation, or 30 mg/kg previous to 10 Gy fragment. Local hyperthermia, for 30 min at 43 °C, succeeded the acute RT or the first 10 Gy fraction. WFA, hyperthermia, and radiation administered separately or in bimodality therapy in melanoma did not generate a complete response (CR). Several CR were identified in fibrosarcoma to enhance following bimodality therapies. The synergistic rise after trimodality therapy of CR was up to 37% in melanoma and up to 64% in fibrosarcoma. Fractioned radiotherapy (10 Gy x 5) was more efficient (25% CR) on melanoma than acute 50 Gy (0% CR), whereas fibrosarcoma response showed no variation between the two procedures. WFA along with fractionated radiotherapy determined a synergistic rise in CR for both tumors; this effect is further amplified by hyperthermia. It is important to analyze WFA’s usefulness in increasing the therapeutic reaction of radiation-resistant tumors against fractionated radiotherapy [39]. Some studies have reported the inhibition of in vivo development of multiple tumor xenografts including uveal melanoma and the sensitizing effect of administering WFA on B16F1 melanoma cells to radiotherapy [36,40]. The WFA therapy was found to induce TRIM16 mRNA expression in melanoma cell lines, while TRIM16 was expected to trigger the highest cytotoxic action. MelCV melanoma cells, compared to MelJD cells, were shown to be less susceptible to WFA therapy. The TRIM16 basal expression of the MelCV cells was lower than that of the MelJD cells [41]. It is believed that, as a result of preexisting lower basal TRIM16, MelCV cells will intrinsically be less susceptible to WFA therapy, and the apoptotic activity of TRIM16 in these cells can be inhibited in different ways. It is not clear how TRIM16 expression is lost in melanoma cells.

Several factors were identified as triggers for neuroblastoma, such as promoter methylation and decreased protein stability; comparable imbalances may appear in melanoma [25]. The induction of TRIM16 mRNA expression at increasing WFA concentrations was shown to be modest, indicating that other regulatory mechanisms, such as post-translational changes, that enhance the stability of TRIM16 or prevent its proteasomal degradation, may also be determined by WFA. Studies showing that WFA may inhibit proteasomal degradation, in the site of TRIM16, sustained the above hypothesis [23]. Studies on migration consistent with research on breast cancer cell lines have shown that WFA prevented melanoma cell migration. Therefore, it is implied that WFA therapy, together with TRIM16 expression upregulation, can be a possible way of preventing disease development and serving as a support therapy for patients with stage II melanoma [42].

## 4. Withaferin Actions in Other Skin Diseases

### 4.1. In Scleroderma

Scleroderma, known also as dermal fibrosis, is considered as a connective tissue autoimmune disorder, having an unidentifiable etiology; it is highly heterogeneous and has various and numerous clinical manifestations [2,3]. Due to this clinical heterogeneity, it is very difficult to establish optimal management of the disease, in terms of an efficient treatment [3,43,44].

This autoimmune condition starts with inflammation due to tissue wounds and gradually accumulates an extracellular matrix, leading to scars and hardening of the skin. Inflammation is a protective reaction to tissue damage determined by many factors. Although inflammation is necessary for curing wounds, irregular chronic inflammation usually determines the scarring of the tissue [45]. The essential role of inflammation in physiology and pathology determines bidirectional effects; inflammation is a protective reaction to tissue damage determined by many factors [46].

The function of WFA was explored in a 28-day murine model of bleomycin-induced experimental scleroderma [47]. WFA was given intraperitoneally, once daily, for 28 days in two doses of 2 and 4 mg/kg of mouse (male C57BL/6 mice, aged 8–9 weeks). A significant decrease in dorsal skin thickness was found at the end of the study. Obtained experimental data show that WFA significantly inhibited proinflammatory fibrosis stages, transforming growth factor (TGF)-β/Smad signaling, and fibroblast conversion into myofibroblasts. Moreover, results show that WFA regulates FoxO3a-protein kinase B (PKB, Akt)-dependent mammalian NF-κβ proteins family/IKK-mediated inflammatory process that is the main signaling route in fibrogenesis. This study indicates WFA as a therapeutic antifibrotic agent in scleroderma [47].

### 4.2. In Disorders of Pigmentation

Pigmentation disorders are recognized as the third most common disorders among dermatologic diseases, being also considered as causing relevant psychosocial impairment [48]. Hyper-/hypopigmentation are considered pigmentation disorders, primary/secondary to other types of diseases. Melasma, postinflammatory hyperpigmentation, ephelides (freckles), café au lait macules, and solar lentigines are representative hyperpigmentation impairments. Although these disorders are usually benign, they may cause discomfort to patients. Adequate dermatologic record, skin analysis, and biopsy, when necessary, may serve to eliminate melanoma and its precursors [49].

Current and future medicine certainly involves the use of revolutionary techniques that optimally manage aesthetic aspects (i.e., topical agents, cryotherapy, laser or light therapy, chemical peeling, or a combination of these techniques [50]). Laser therapy or surgical excision can be used to treat café au lait macules if the patient desires. Postinflammatory hypopigmentation, white pityriasis, vitiligo, and tinea versicolor are considered impaired hypopigmentation.

Determined by the spread and development on the skin, vitiligo therapy involves treatment with ultraviolet A (regardless of the presence of psoralens), cosmetic coating and topical corticosteroids, treatment with ultraviolet B (narrowband), and calcineurin inhibitors. Patients presenting self-limited stable vitiligo can be subjected to grafting procedures, while the ones having considerable impairment can undergo depigmentation treatment in order to uniformize their skin tone. Treating the underlying disorder may heal or ameliorate other hypopigmentation conditions [51,52].

#### 4.2.1. Hyperpigmentation

It has been shown that redox imbalances are closely connected to a wide range of changed cellular reactions and have an important impact on intracellular signaling routes, particularly the protein kinase C/mitogen activated protein kinase (PKC/MAPK) route, an important pathway that controls melanogenesis in human melanocytes [53].

In order to understand the role of redox balance adjustment action on epidermal hyperpigmentation conditions, an antioxidant-rich herbal extract of WS was used to evaluate the effect on endothelin 1 (EDN1) stimulated pigmentation and to analyze the biological mechanisms. An important depigmentation effect on EDN1 (10 nm)-induced pigmentation, associated with a major decrease of eumelanin amount was obtained by adding the *WS* extract (10 μg/mL) [54]. The reverse transcription–polymerase chain reaction analysis (RT–PCR) and Western blotting highlighted a significant suppression of the stimulated expression of melanocyte-specific mRNAs and proteins, and a microphthalmia-associated transcription factor (MITF) at days 7–10 of culture, with *WS* extract (10 mg/mL) indicating a deterioration of intracellular signaling upregulation. Signaling experiments have shown a significant deficiency in endothelin (EDN)-1 (10 nm)-induced phosphorylation of Raf-1, MEK, ERK, MITF, and cyclic AMP responsive element-binding protein (CREB) after 15 min from EDN1 treatment in *WS* extract (10 μg/mL) treated human melanoma cells in culture. WFA therapy, involving administered concentrations of 10–50 μm, determined considerable downregulation of EDN1 induced phosphorylation of Raf-1, MEK, ERK, MITF, and CREB after 15 min from EDN1 administration [54].

#### 4.2.2. Hypopigmentation (Leucoderma)

A type of smooth muscle cell in isolated skin melanophores of *Rana tigerina* frog, treated with *WS* root extracts along with pure WFA, provides excellent opportunities for in vitro investigation of the effects of pharmaceutical substances and pharmacotherapy. *WS* lyophilized extract and its active ingredient WFA greatly influenced dose-dependent, physiologically significant melanin distribution activity in *R. tigerina* isolated skin melanophores that were completely inhibited by hyoscine and atropine. The lyophilized *WS* extracts and their active ingredient WFA effects on melanin distribution were considerably enhanced by neostigmine. The effects of the extracts of *WS*, containing WFA, on melanin distribution seem to be mediated by cholino-muscarinic-like receptors presenting similar characteristics [55].

### 4.3. Viral Infections

Given the large number and diversity of dermatological diseases, viral disorders of this organ are a significant part, most of them manifested by a rash (exanthem) and, in many cases, accompanying lesions involving the mucous membrane (accompanying enanthema). All of these can affect patients of any age and can vary widely, including both complex systemic diseases and simple superficial rashes [56].

#### 4.3.1. Herpes Simplex

Herpes simplex virus of type 1 and 2 (HSV-1/HSV-2) are part of the Herpesviridae family, being reported that approximately 85% of the population was infected with at least one of them [2]. These two viruses are known to trigger diseases, some of which are mild (corneal eye, mouth cold sores, or genitals lesions, etc.), and some are among the most serious, even fatal (fatal herpes encephalitis) [3]. Obviously, those subjects who have a weakened or suppressed immune system (i.e., HIV-infected patients) are more likely to be infected with HSV [4,57,58,59].

Simulated experiments (docking, molecular dynamics simulation models) were carried out to study the binding mechanism of prospective WFA herbal drugs on the composition of DNA polymerase of herpes simplex virus. The simulation results reveal great affinity in the binding of the ligand to the receptor, findings of the docking simulations having a high ligand-receptor affinity [58,59].

Long de novo molecular dynamics (MD) simulations conducted for 10 ns helped in assessing the system dynamic behavior to confirm the dockage findings and determine the key residues in the enzyme-inhibitor interactions. The established MD models are based on the premise that WFA is a possible ligand to target and inhibit herpes simplex virus DNA polymerase. The study findings also direct the design of highly specialized and effective DNA POL selective inhibitors and powerful activity in order to expand the available therapeutic tools against the biologically hazardous warfare agent of herpes simplex virus [60].

#### 4.3.2. Papilloma Virus

HPV infection is defined as a viral infection that usually causes abnormal growths in the skin or/and mucous membranes, being known >100 human varieties. Depending on the type of HPV contacted, it can cause various manifestations, such as warts, various types of cancer, etc., mainly infecting the differentiated squamous epithelium. It should be noted that in humans, almost every part of the skin can be infected [61,62]. 

In mice models, Ashwagandha plant extract blocked benzo(a) pyrene-induced forestomach papilloma genesis, carrageenin-induced air pouch granuloma, and DMBA-induced skin papilloma genesis with 60 to 92% and 45 to 71%, respectively, inhibition in tumor occurrence and multiplicity. *WS* prevents the formation of skin papilloma caused by 7,12-dimethylbennzanthracene. However, this plant seems to have no toxic impact on mice during the trial [63].

## 5. Discussion

Leaves, flowers, roots, stem, and bark of the WS plant are recognized for their ability to cure/ameliorate multiple disorders (i.e., heart, liver, respiratory, sexual, tumor, wounds/ulcers, etc.) or symptoms (inflammation, fever, etc.) [64]. The published data proved that withanolides are the ones to which all these benefits mentioned above are attributed [65].

The extracts obtained from different parts of WS are used in a diverse range of cosmetic formulations and food supplements, presented as having numerous benefits for skincare, from their calming, protective, regenerating, and revitalizing effects to their use as UV protection screen and venotonics [66,67].

Table 1 and Table 2 present the pharmaceutical/cosmetic products containing WS extracts available for the population and the summarized results of studies that have investigated the effects of WS in certain dermatological diseases [66,67].

In addition, Ashwagandha roots are considered and recognized to have an ameliorating effect in the treatment of leukoderma, ulcers, scabies, etc.; moreover, when applied topically, they have a healing effect on skin wounds and diminish swelling [8].

These results imply that one or several Ashwagandha elements present physiological activity on the skin. Furthermore, the boiled mixture of Ashwagandha roots and leaves is believed (in Ayurveda) to present healing properties in treating wounds [18]. There was no scientific approach to evaluate whether this treatment is efficient. Extrapolating the proper investigations, the outcome can be expended to WFA offering the reason to apply WFA treatment in other dermatological pathologies than those presented in this review.

Different from the majority of synthetic medicines created to present increased selectivity and decreased side effects, WFA is a natural product concomitantly targeting more proteins. This multitargeting perspective could be favorable for the treatment of disorders generated through anti-inflammatory mechanisms in which a disequilibrium among several pro-/anti-inflammatory factors is present [68,69,70]. Most chronic skin diseases with increasing incidence (acne, dermatitis, psoriasis, etc.) have an inflammatory component.

The effects of WA could be investigated in other autoimmune diseases, epidermo-neuroviruses, pigmentation disorders (melasma), or in several antiaging preparations. WFA therapy could improve or potentiate the associated dermatological therapies, helping the patient’s favorable course in chronic diseases. The chemopreventive action and the melanin regulatory activity of WS aqueous and organic extracts used as a topical treatment for skin cancer were demonstrated by several studies [17]. The formulation and testing of topical preparations with WFA in various skin conditions would also be necessary in order to determine the antiproliferative, antioxidant, immunostimulatory, or antiaging effects [71,72,73,74].

On the other hand, although several in vivo results highlight the chemopreventive effect of WA, there are only two studies that have demonstrated this type of effect of WA on skin cancers, although high protection was demonstrated against tumor formation. Assessing the effectiveness of WFA in chemoprevention in a wide variety of animal cancer models may enhance the possible WS contribution in preventing carcinogenesis. There were no clinical studies with WFA on humans with cancer or cancer biomarkers as outcomes. WS effect, however, was evaluated in some clinical studies on different conditions and impairments. Although numerous assessments had important disadvantages (i.e., being limited to reduced sample size, using compound combinations, taking old subjects as case studies), the results represent a basis for further translational and clinical studies on WFA properties.

## 6. Conclusions

Following this analysis, it is clear and obvious that WFA has appreciable potential as a future “key-role player” that can be integrated into therapies/treatment of dermatological diseases. Our study reviewed the results of numerous studies on the topic, published to date, and focused on the role of WFA in the treatment of some skin diseases.

These published data are extremely encouraging and take into account the fact that WFA needs to be studied in detail, in order to discover and observe other potential therapeutic effects. Certainly, the use of WFA (both alone and in combination with other phytochemicals or drugs), in large-scale clinical trials for a variety of skin conditions, remains to be studied.

## Figures and Tables

**Table 1:** Actions of the cosmetics and food supplements containing WS extracts on the skin [66,67].

Active Substances		Product		Actions
*WS* root extract	300 mg	Capsules		Has a general rejuvenating effect
	500 mg			Antioxidant, anti-aging
*WS* extract (unmentioned origin)		Cream	Body	Protects the skin against pollutants and dry climate
			Face	Nourishing, antioxidant, and rejuvenating
			Skincare	Moisturizes the skin and soothes irritated skin
		Oil	Baby body	Improves skin tone, protects against dehydration and harmful external factors
			Massage	Strengthens, giving vitality and increasing the resilience of the skin
			Soap	Toning effect, for stressed and exhausted skin
		Shower gel		Toning effect
*WS* extract ENERGINIUS™		Cream		Restore skin vitality; balance fatigue generated by digital pollution (time lost in the front of monitors,) environmental pollution, etc.

**Table 2:** *WS* root/seed extract usage in dermatological diseases and its actions and results.

Dermatological Disorder	Actions/Results of WS/WFA	Ref.
Carcinoma	*WS* roots demonstrated a 49% inhibitory effect on CHO cells colonization capacity. Cell development and adhesion are inhibited by WS, which induces long-term inhibition of CHO cell development, depending on cell density and length of Ashwagandha subjection. This information helps oncologists who intend to use Ashwagandha as synergizer, in completion to traditional therapies—radio- and chemotherapy.	[64]
Human malignant melanoma	*WS* plant extracts have anticancer properties, resulting in that fresh aqueous extract enhanced the cytotoxic effect correlated to melanoma (human malignant A375 cells).	[26]
Hypomelanosis	*WS* extract reduces endothelin-1-induced pigmentation in human epidermis by impeding the PKC action at the melanocytes level.	[43]
Hypopigmentation	*WS* and its natural chemicals *(WFA* and *Astaxanthin)* are the newest suggestions for possible anti-pigmenting substances, thus avoiding hypopigmentation risks.	[65]
Psoriasis Skin inflammation	WS was demonstrated to contain favorable fatty acids by the GC-FID analysis, while HPLC analysis revealed a small quantity of withanolides in WS. The WS seed * fatty acids diminished psoriatic wounds and skin inflammation in TPA-triggered, psoriatic-like mouse model. Research based on the study of TPA- or LPS-induced cells showed considerable anti-inflammatory action of WS in adjusting NFκB action and reducing the discharge of pro-inflammatory cytokines, IL-6, and TNF-α. Associating the skin reparatory and anti-inflammatory properties, WS seed fatty acids have powerful antipsoriatic action.	[66]
Scleroderma	It is suggested that WFA could repress the pro-inflammatory stage of fibrosis, TGF-β/Smad signaling, and considerably inhibit fibroblast conversion to myofibroblasts. Furthermore, it was revealed that the main signaling route in fibrogenesis (FoxO3a-Akt-dependent NF-κβ/IKK-mediated inflammatory response) is modulated by WFA. The results present WFA as an antifibrotic factor having favorable activity in scleroderma.	[38]
Skin rejuvenating agent	*Ashwagandha* has rejuvenating action and growth-promoting activity, both being evaluated in a 60-day study on 60 children in good health. Findings show that it can be administered as a hematinic and growth promotor in children. Medical research using root extracts of *Ashwagandha* shows that it presents considerable antiaging activity in healthy aged subjects.	[67]

* Seeds extract. Legend: *WS*—*Withania somnifera;* WFA—withaferin A; TPA—12-O-tetradecanoylphorbol 13-acetate; NFκB—nuclear factor-Κb; TGF-β—transforming growth factor β; Smad—small mother against decapentaplegic; IKK—IκB kinase; IL-6—interleukin-6; CHO cells—Chinese hamster ovary cells; TNF-α—tumor necrosis factor α.
